# Lymphoproliferative Lesions in the Setting of HIV Infection: A Five-Year Retrospective Case Series and Review

**DOI:** 10.4061/2011/618760

**Published:** 2011-03-30

**Authors:** Etienne Mahe, Catherine Ross, Monalisa Sur

**Affiliations:** Division of Anatomical Pathology, Department of Pathology and Molecular Medicine, Juravinski Hospital and Cancer Centre, McMaster University, 711 Concession Street, Hamilton, ON, Canada L8V 1C

## Abstract

A wide variety of noninfectious lesions have been identified in association with HIV infection. Many hematolymphoid lesions are possible in this patient group, both reactive and neoplastic. Epidemiologic data suggests that lymphoid malignancies are among the most common neoplasms in patients with HIV. We present a selective case series assembled over a 5-year period from the relatively low HIV-prevalence Hamilton Regional Laboratory Medicine Program (HRLMP), a tertiary care referral centre in Southern Ontario. This series serves to demonstrate the wide variety of lymphoid lesions that may be encountered in patients with HIV. In addition to outlining the pathologic work-up necessary in these cases, we discuss characteristics that distinguish the HIV-associated lesions from the pathobiologically similar non-HIV-associated lymphoid lesions.

## 1. Introduction

The HIV/AIDS epidemic bears the dubious distinction of one of the world's most devastating yet medically challenging disease entities. Despite the rapidity of important advances in HIV/AIDS therapies over recent decades and despite the countless billions of dollars invested in research and development, a cure continues to elude the scientific community. So globally profound is the HIV/AIDS epidemic that, according to the most recent UNAIDS epidemiology update, the global total of HIV-positive persons is nearly equal to the total population of Canada [1, 2].

Although most of the mechanisms leading to death from HIV infection are related to immunodeficiency, neoplasia has long been recognized as a major contributor to morbidity and mortality of the HIV/AIDS population [3]. Many of the AIDS-related neoplasms are hematolymphoid; indeed, AIDS-related lymphomas, in some current case series, have superseded Kaposi's sarcoma as the commonest AIDS-related neoplasm [4].

HIV infection has been calculated to increase the likelihood of developing a lymphoma by 60–110 times [5, 6]. As noted by the World Health Organization (WHO), HIV/AIDS-related lymphomas are predominantly B-cell lymphomas [6]. A number of the common HIV-related B-cell lymphomas are AIDS-defining illnesses [7]. It must be noted, however, that a notable number of aggressive peripheral T-cell lymphomas have been recognized in HIV-positive individuals. The three most common lymphomas associated with HIV are Burkitt's lymphoma, diffuse large B-cell lymphoma, and Hodgkin's lymphoma as currently defined in the 2008 WHO classification [6]. 

As a lentivirus, HIV shows a striking tropism for CD4 T-lymphocytes as well as dendritic cells and monocytes. The virus exploits the host CD4 cell chemokine receptors to gain access into the host cell wherein it self-incorporates into the host cell genome, and engages in self-replication. This tropism for one of the human immune system's major immune response modulators is believed to be the main pathophysiologic mechanism leading to AIDS; the result is a critically reduced number of CD4+ cells, which in turn perturbs the intricate CD4-dependent host immune response. In addition, its tropism for the constituent cells of the lymph node serves to concentrate the immunologic response in the lymph nodes, resulting in a variety of pathologic and potentially neoplastic changes [8].

The current paradigm suggests that it is the abnormalities of the host immune response, and not direct oncogenesis, that lead to the markedly increased HIV-related risk of lymphoproliferative disease. Epidemiologic studies have observed lower incidences of HIV-related lymphomas in patients receiving HAART relative to those that do not [9, 10]. Other studies noted that patients with certain HIV-related lymphomas receiving HAART showed a relatively improved prognosis than their untreated counterparts [11, 12]. Furthermore, the wide variety of lymphoid malignancies associated with HIV argues against a direct oncogenic influence. Other studies have noted that HIV DNA, unlike some other known oncogenic viruses, does not seem to incorporate itself at specific locations in the host cell genome [13, 14] while other studies examining the gene expression patterns in AIDS-related malignancies have noted that, in the majority, HIV DNA or proteins could not be identified in malignant cells [15]. 

The combined immunosuppression and immunologic stimulation present in HIV infection may synergize with other infectious agents known to be oncogenic, such as EBV, to produce lymphoproliferative disease. This is consistent with the observation of EBV positivity in greater proportions in HIV-associated hematolymphoid lesions than in those in the non-HIV population.

In the local Health Integration Network served by HRLMP, all known HIV-positive patients are diligently followed by the regional Special Immunology Services clinic. Recent (2008) census data estimates a relatively low HIV/AIDS prevalence of 91 per 100000 in the Hamilton region [16, 17]. The HRLMP receives all tissue specimens pertaining to HIV-positive patients in the region. We performed a 5-year retrospective review of all pathology reports and identified 12 cases with an HIV-associated lymphoproliferative disorder (Table 1). The following explores the relevant clinical and pathological features of these lesions, highlighting the distinguishing features they may have relative to their non-HIV associated kindred lesions, and some of the difficulties and pitfalls in diagnosis.

## 2. HIV-Associated Lymphadenitis

HIV-associated lymphadenitis is very common in HIV-infected individuals and was identified as a complication of HIV infection very soon after the HIV/AIDS epidemic was identified [18]. Lymphadenitis often accompanies the acute phase of HIV infection, usually accompanied by brief (sometimes barely noticeable) flu-like symptoms [19]. Even as the acute phase of HIV infection wanes, the associated lymphadenitis can be markedly persistent [19]. This is likely due to the profound lymphotropism previously described [8]. In cases of profound immunodeficiency, lymphadenitis due to concurrent infection by mycobacteria or fungi may confuse the diagnosis [20]. Clinical concern of a lymphoproliferative disorder may also be raised in cases of persistently enlarged groups of lymph nodes or in cases of suddenly enlarged nodes in patients for whom a concomitant infectious etiology has been ruled out. These clinical scenarios are more frequently encountered in the HIV positive population in Ontario in whom TB and other infections are infrequent.

**Figure 1:**
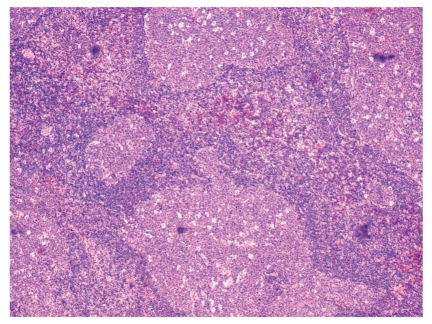
HIV-associated lymphadenopathy with hyperplastic follicles taking on atypical shapes (hematoxylin and eosin, 100x).

The histologic features of HIV-associated lymphadenitis follow a well-characterized pattern of histomorphological evolution [8]. In Grade 1, usually seen early on in the course of HIV infection, the lymph nodes display exuberant hyperplastic features. Irregular enlargement of germinal centres is noted, often with a prominent starry sky pattern due to increased germinal centre cell apoptosis and subsequent phagocytosis of cellular debris by tingible-body macrophages (Figure 1). In addition, there may be focal areas of expansion of the interfollicular zones by monocytoid cells (transformed B lymphocytes) with an accompanying reduction of mantle/marginal zone lymphocytes. The exuberance with which the latter occur leads to a distinct form of “follicular lysis” producing atypical convoluted germinal centres (Figure 1). Scattered multinucleated giant cells and neutrophils may also be identified. In Grade 2, the key features are reduced lymphoid follicles with a relative reduction in the number of mature lymphocytes. An increased number of plasma cells will be noted, in addition to a proliferation of perifollicular blood vessels. As Grade 2 evolves into Grade 3, the residual germinal centres become sclerotic. In some cases, a Castleman-like pattern of hyalinized germinal centres may be observed; Castleman's disease is generally a non-HIV-related disorder exhibiting a similar pattern of germinal centre sclerosis in lymph nodes.

The patterns of HIV lymphadenitis have been identified in other non-HIV-related conditions and are therefore not specific to HIV. When identified in the correct clinical context, however, they are reproducible and may offer valuable clinical information relating to the natural history of HIV infection. Grade 3, for instance, is frequently seen in HIV patients with AIDS [21]. Kaposi's sarcoma is frequently associated with Grade 3 lymphadenitis [8]. Most importantly, a statistically significant survival reduction has been identified as HIV-associated lymphadenitis evolves from Grades 1 to 3 [21]. In addition to the recognition of the Grades of HIV-associated lymphadenitis, it is incumbent on the surgical pathologist to diligently rule out a lymphoproliferative disorder in suspicious lymphadenopathy. The latter is of particular importance in cases in which atypical lymphoid cells are recognized in an otherwise typical background of HIV lymphadenitis. 

**Figure 2:**
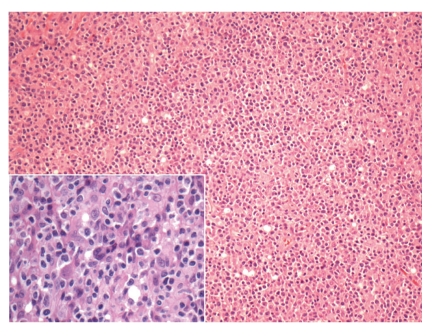
HIV-associated lymphadenopathy; lymph node demonstrating reactive features (hematoxylin and eosin, 100x); inset shows atypical EBV-infected lymphoid cells (hematoxylin & eosin, 200x).

Epstein-Barr virus (EBV) seroconversion is very common in the population at large, and in the HIV-positive population specifically. Concomitant benign EBV-associated changes in a lymph node may produce atypical Reed-Sternberg-like cells in a polymorphous background, as seen in Figure 2. A basic immunohistochemical panel is often required to identify the lesion as reactive. The prototypic immunoprofile of the reactive lymph node should be present: CD3 will highlight the mature T-cells predominant in the interfollicular zones; CD20 will highlight the reactive B-cells and immunoblasts of the interfollicular zones; BCL-2 will highlight the extragerminal centre lymphocytes of a reactive lymph node whilst BLC-6 and CD10 will be positive within a reactive germinal centre; CD23 will highlight the follicular dendritic cell meshwork of germinal centres (a helpful marker in cases of questionable follicular lysis); Ki-67 will show high proliferation index restricted to the germinal centres. In cases in which atypical cells are identified, as in the Reed-Sternberg-like cells of Figure 2, a combination of LCA, CD20, EBV, CD15, and CD30 will help to rule out Hodgkin's lymphoma; the atypical Reed-Sternberg-like cells will be positive for LCA, CD20, EBV, with or without positivity for CD30 (a cellular activation marker), and negative for CD15. EBV testing (either as immunohistochemistry latent-membrane protein staining or by means of in situ hybridization) is very helpful in cases of lymphadenitis with atypical cells. In cases of Grade 2 or 3 HIV-associated lymphadenitis, wherein the lesions are lymphocyte depleted and may show a proliferation of fibroblasts and germinal centre sclerosis, immunohistochemistry for HHV-8 will help rule out Kaposi's sarcoma. The latter entity is known to be associated with higher-grade HIV-lymphadenitis [21].

## 3. Polymorphic Lymphoid Proliferations Resembling Posttransplant Lymphoproliferative Disease (HIV-PLP)

The WHO makes brief reference to this relatively poorly defined entity, occurring in less than 5% of HIV patients [6]. In our health region, this entity has been encountered only once in the past five years. It is mentioned with precedence in this review given the unique approach to treatment that its diagnosis necessitates. Specifically, given that the malignant nature of HIV-PLP remains in doubt [22–24], therapy for these patients in our institution typically focuses on immunomodulation and not chemotherapy. The rarity of these lesions, however, precludes rigorous trials exploring different treatment regimens, and the current state of the art relies heavily on inferences made from the more thoroughly studied posttransplant lymphoproliferative diseases. Currently, the limited available evidence suggests that cases of HIV-PLP likely represent a spectrum of entities spanning the reactive to the malignant [22]. In the largest case series to date, Nador et al. noted that the vast majority of cases of HIV-PLP did not show the gene rearrangements of B-cells common to other malignant lymphomas [22]. Also, unlike most HIV-associated lymphoproliferative disorders, HIV-PLP patients seem to present at a relatively low stage [22]. 

**Figure 4:**
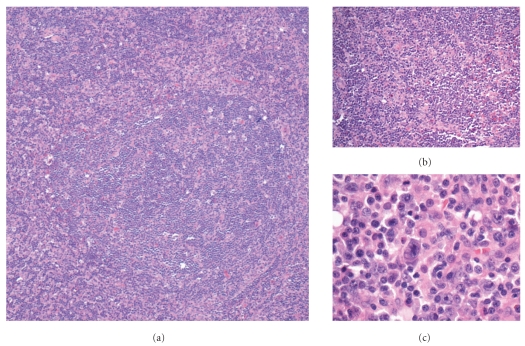
PTLD-like polymorphous B-cell lymphoproliferative disorder; (a) follicular lysis (hematoxylin and eosin, 100x); (b) polymorphous inflammatory infiltrate (hematoxylin and eosin, 100x); (c) atypical activated lymphocytes (hematoxylin and eosin, 400x).

Not surprisingly, the histomorphology of HIV-PLP closely resembles posttransplant lymphoproliferative disease. There may be a striking polymorphous infiltrate of small lymphocytes, plasma cells, immunoblasts, histiocytes, and eosinophils. In the single case encountered in our institution over the course of the previous five years, prominent reactive lymphoid follicles with areas of follicular hyperplasia and follicular lysis were encountered (Figure 4(a)). A few scattered large atypical cells were also identified (Figures 4(b) and 4(c)), though these were not classic Reed-Sternberg cells.

Most HIV-PLP are predominantly B-cell proliferations and thereby show strong staining for CD20 and CD79a. A background of reactive CD3-positive T-cells will be seen, intermixed with other inflammatory cells. To further classify the lesion, a broad panel of immunomarkers may be required; we recommend a preliminary panel including CD10, BCL-2, BCL-6, CD15, and CD30. Flow cytometry may be helpful in identifying the presence of monoclonal B-cells, should they be present. At our institution, we also employ polymerase chain reaction testing for B-cell gene rearrangements to assess for the presence of a monoclonal B-cell population. In contrast to the monomorphic HIV-PLP, the polymorphic variants may or may not demonstrate unequivocal monoclonal B-cell populations. These cases need to be assessed and diagnosed in the proper clinical settings. Testing for EBV, either by latent membrane protein immunohistochemistry, or by means of the more sensitive in situ hybridization, is also indicated since a number of HIV-PLP are EBV positive [22].

## 4. HIV-Associated Burkitt's Lymphoma (HIV-BL)

In stark contrast to HIV-PLP, HIV-BL is much more common and much more aggressive. Indeed, Burkitt's lymphoma is 1000-times more common in HIV patients than the general population and accounts for up to 40% of HIV-associated lymphomas [6, 25]. HIV-BL also presents relatively early on in the course of HIV infection and, as may be seen in the general population as well, at a relatively young age [26]. Patients with HIV-PL will often present with nodal disease but, relative to non-HIV-associated Burkitt's lymphomas, more frequently demonstrate extra-nodal involvement [27]. 

**Figure 5:**
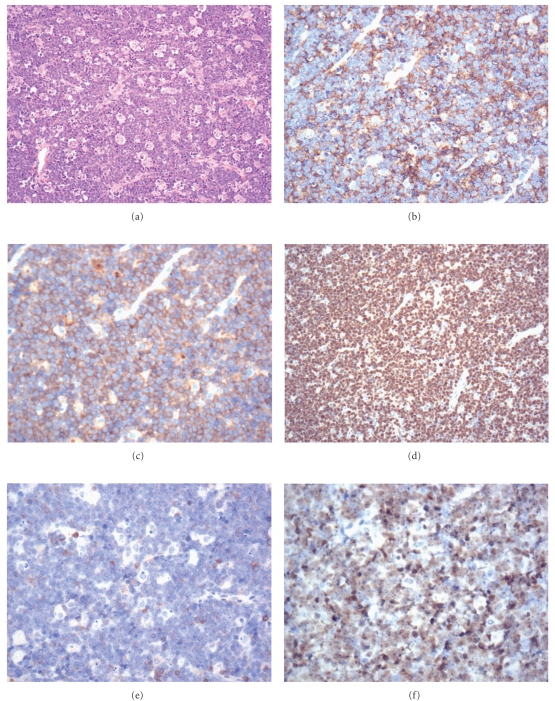
Burkitt's lymphoma; (a) prominent “starry-sky” appearance (hematoxylin and eosin, 100x); (b) CD20 stain (100x); (c) CD10 stain (100x); (d) almost 100% Ki67 staining (100x); (e) BCL-2 stain (100x); BCL-6 stain (100x).

The histological features of HIV-BL are identical to those seen in other Burkitt's lymphomas. Low power examination typically reveals an expansile lesion diffusely effacing normal tissues (both nodal and extra-nodal). Interspersed amongst intermediately (~12 *μ*m) sized cells with basophilic cytoplasm and oval nuclei with distinct nucleoli, are macrophages with tangible bodies, imparting the classically described starry-sky appearance on low power. Mitoses and apoptotic debris are commonplace.  Figure 5 demonstrates a classic Burkitt's morphology encountered in an HIV patient with involvement of liver and right adrenal. The immunophenotype of HIV-BL is also similar to other Burkitt's lymphomas. Burkitt's lymphoma cells are LCA, CD20, CD79a, PAX5, CD10, BCL-6, and EBV positive and negative for BCL-2, CD3, CD5, CD21, CD23, CD43, cyclin D1, and TdT. HIV-BL also shows a virtually 100% Ki67 proliferation index. Studies have indicated that the plasmacytoid variant is most common in HIV-BL, often highlighted with CD138 staining [27]. Molecular testing in HIV-BL often demonstrates the characteristic Burkitt's C-MYC (t(8;14)) mutation; this mutation aligns the oncogenic MYC region with the transcriptionally active immunoglobulin loci on chromosome 14.

Treatment regimens for HIV-BL have changed since the introduction of highly active antiretrovirals. Prior to HAART therapy, HIV-BL was treated with the standard cyclophosphamide, hydroxydaunorubicin, vincristine, and prednisone (CHOP) chemotherapy (the mainstay therapy for HIV-associated diffuse large B-cell lymphoma) with mean survival of only 6 months [27, 28]. The literature currently advises against the CHOP approach in HIV-BL based on evidence that these patients fare poorly relative to CHOP-treated cases of HIV-associated DLBCL [27, 28]. This fact serves to underscore the weight that a correct diagnosis of HIV-BL relative to other HIV-associated lymphomas bears. Current regimens, such as that employed at our institution, focus on relatively aggressive therapies including combinations of cyclophospamide, doxorubicin, vincristine, and methotrexate. Trials assessing the potential benefit of rituximab are ongoing [27].

## 5. HIV-Associated Hodgkin's Lymphoma (HIV-HL)

Intriguing observations regarding the incidence of HIV-HL have been made, namely, that the incidence of HIV-HL increases with an HIV patient's CD4 count [29]. The incidence of HIV-HL, however, remains low relative to the other more common HIV-associated lymphomas (namely, HIV-DLBCL and HIV-BL). It has been suggested that the combination of improved CD4 counts in the context of HIV infection causes a relatively enhanced cytokine milieu, serving to stimulate the survival and proliferation of neoplastic Reed-Sternberg cells [30]. The relative incidence of the variants of HIV-HL is also intriguing, with the mixed cellularity variant notably more common than the nodular sclerosing, in contradistinction to the non-HIV-associated Hodgkin's lymphoma [29].

**Figure 6:**
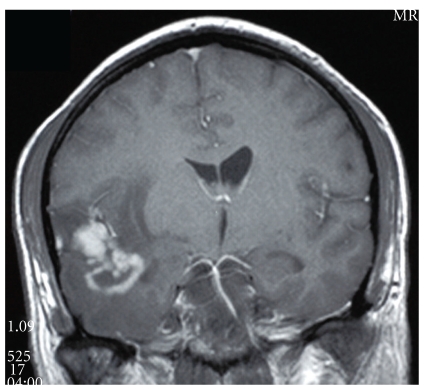
Central nervous system Hodgkin's lymphoma. Computed tomography image demonstrating an ill-defined ring-enhancing intracranial lesion with peritumoural edema.

**Figure 7:**
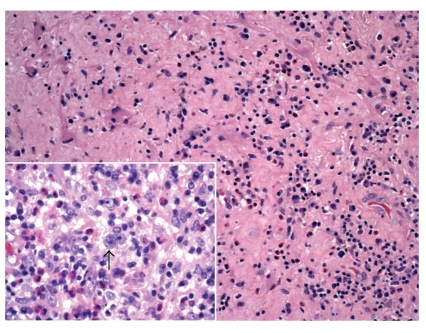
Central nervous system Hodgkin's lymphoma. Brain tissue with polymorphous inflammatory infiltrate (hematoxylin and eosin, 100x); inset: arrow highlights Reed-Sternberg cell with polymorphous inflammatory background (hematoxylin and eosin, 400x).

The morphologic features of HIV-HL are consistent with those of non-HIV-associated Hodgkin's lymphoma. In the more common mixed cellularity subtype, a polymorphous background of histiocytes, eosinophils, plasma cells, neutrophils, and benign small lymphoid cells may be seen. Scattered in this infiltrate will be the large atypical binucleate Reed-Sternberg or the mononucleate Hodgkin's cells, sometimes surrounded by a rosette of small lymphocytes. As in the non-HIV-associated Hodgkin's cases, classic Reed-Sternberg or Hodgkin's cells are required; these should be large, 20–60 *μ*m, with eosinophilic to amphophilic cytoplasm, bearing mirror image nuclear lobes with inclusion-like eosinophilic nucleoli in the case of Reed-Sternberg cells or mononucleate with large eosinophilic inclusion like nucleoli in the case of Hodgkin's cells. Figures 6 and 7 demonstrate an intriguing case of CNS HIV-HL encountered in our institution. In this case, the correct diagnosis was delayed given that the initial stereotactic brain biopsies did not demonstrate Reed-Sternberg cells and showed a polymorphous inflammatory background with epithelioid granulomas, raising the possibility of an infectious etiology. The correct diagnosis was arrived at after receipt of a subsequent resection specimen. This case serves as an example of the need for ample tissue for suspect lymphoproliferative disorders, particularly in CNS lesions which are most commonly stereotactically biopsied for primary diagnosis.

**Figure 8:**
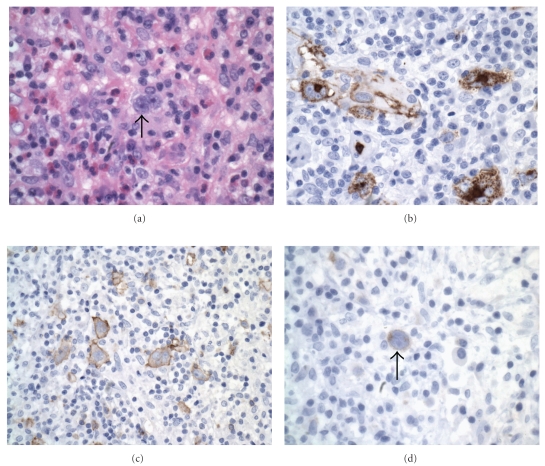
Hodgkin's lymphoma; (a) Hodgkin's cells (hematoxylin and eosin, 400x); (b) CD15-positive Hodgkin's/Reed-Sternberg cells (400x); (c) CD30-positive Hodgkin's/Reed-Sternberg cells (400x); (d) EBV stain positive in Reed-Sternberg cell (400x).

The HIV-HL immunophenotype is identical to that seen in non-HIV-associated Hodgkin's. In cases with the common classical morphology, the Reed-Sternberg or Hodgkin's cells can be highlighted with CD15, CD30, and EBV-LMP staining (Figure 8). These cells are typically negative for LCA, T-cell markers, CD20, CD79a, CD10, ALK-1, and EMA. Flow cytometry, though not of direct diagnostic utility in HIV-HL, can confirm the polymorphous polyclonal background inflammatory infiltrate.

Patients with HIV-HL typically present with more advanced-stage disease relative to non-HIV-associated Hodgkin's lymphoma and B-symptoms are common [28]. Standard therapy for HIV-HL is a combination of optimized HAART with ABVD chemotherapy, as is currently employed in our institution. In addition, several authorities recommend the addition of G-CSF, as many HIV-HL cases may be complicated by pancytopenias [27, 28].

## 6. HIV-Associated Diffuse Large B-Cell Lymphoma (HIV-DLBCL)

A number of distinct epidemiological differences between HIV-DLBCL and non-HIV-associated DLBCL have been noted. DLBCL is relatively less common in the HIV population than in the non-HIV population. In the HIV population, DLBCL is less common than Burkitt's lymphoma [6, 27, 31]. In HIV-positive patients, furthermore, extra-nodal involvement is far more frequent than in non-HIV DLBCL [15]. HIV-DLBCL cases are also more likely to present at advanced stages than non-HIV-associated DLBCL. In our 5-year retrospective institutional review, HIV-DLBCL was identified in only 2 of the HIV-associated lymphomas.

**Figure 9:**
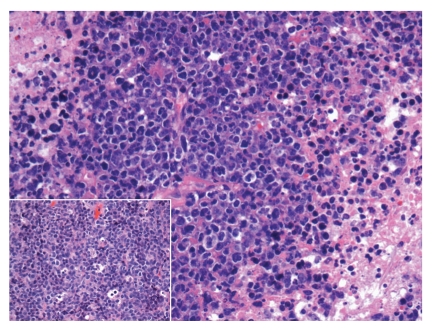
Diffuse large B-cell lymphoma with necrotic background (hematoxylin and eosin, 200x). Inset: large atypical cells (hematoxylin and eosin, 200x).

**Figure 10:**
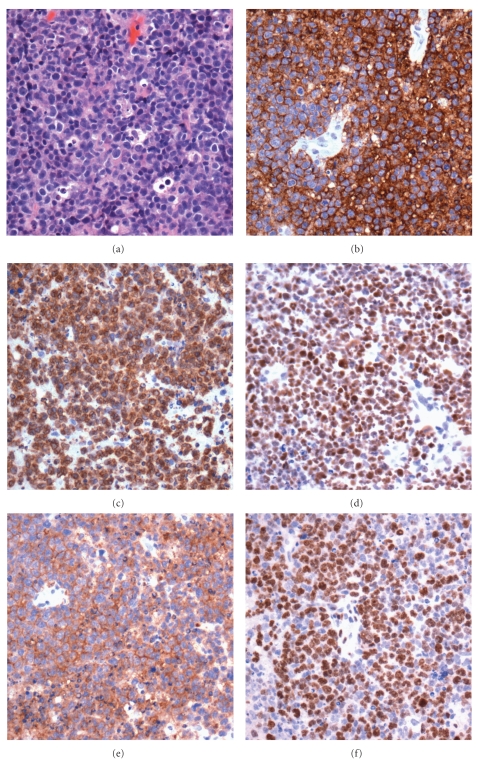
(a) Diffuse large B-cell lymphoma (hematoxylin and eosin, 200x); (b) CD20 stain (200x); (c) BCL-2 stain (200x); (d) Ki-67 stain (200x); (e) CD10 stain (200x); (f) BCL-6 stain (200x).

The histological and immunophenotypic features of HIV-DLBCL are similar to the HIV-negative cases. The architecture of involved tissue, either nodal or extra-nodal, is diffusely replaced by sheets of large lymphoid cells (Figure 9). As in the HIV-negative population, HIV-DLBCL may show a number of morphologic variants; the characteristic HIV-DLBCL variant is the immunoblastic type, in which (as required by the WHO definition), greater than 90% of the neoplastic cells bear a morphology reminiscent of immunoblasts with eccentrically oriented nuclei in basophilic cytoplasm. HIV-DLBCL is positive for pan-B cell markers including CD19, CD20, CD79a, and PAX5. Differing rates of positivity for CD10 and BLC-6 relative to MUM-1 reflect differing germinal centre versus activated B-cell phenotypes [32]. Typical Ki-67 staining ranges from 40–60% (Figure 10). EBV is far more frequently positive in HIV-DLBCL than in non-HIV-associated DLBCL [27].

The diagnosis of HIV-DLBCL has important treatment consequences relative to non-HIV-associated DLBCL. In particular, recent studies looking at the use of rituximab, commonly used in chemotherapy regimens for DLBCL in the non-HIV population, noted an increased treatment-related infection risk [27]. Currently, there are recommendations against treating HIV-DLBCL patients also severely immunocompromised. Radiotherapy is generally not employed in primary therapy in HIV-DLBCL given the frequent advanced stage at presentation [27]. In our institution, CHOP with rituximab is the standard chemotherapeutic regimen with optimization of immunostatus with HAART.

## 7. HIV-Associated Plasmablastic Lymphoma

**Figure 11:**
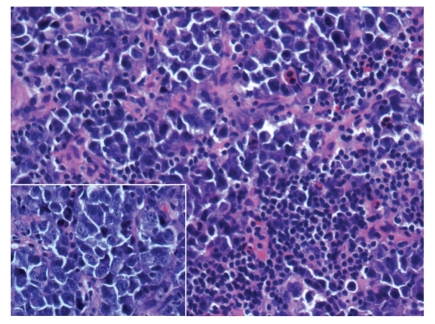
Plasmablastic lymphoma (hematoxylin and eosin, 200x). Inset: plasmacytoid features (large cells with eccentric nuclei, hematoxylin and eosin, 400x).

Plasmablastic lymphoma is a rare entity with characteristic morphologic and immunophenotypic features and is one of the lymphomas originally considered specific to HIV-positive patients [6]. Although it was thought to initially be restricted to the oral cavity, HIV-PBL has been diagnosed at other extra-nodal sites [9], including one noted in the abdominal viscera of an HIV patient in our institution (Figure 11). Some authors consider plasmablastic lymphomas to be a variant of DLBCL [31], though HIV-PBL stands apart given its aggressiveness [6] and can, therefore, be classified distinctly. 

**Figure 12:**
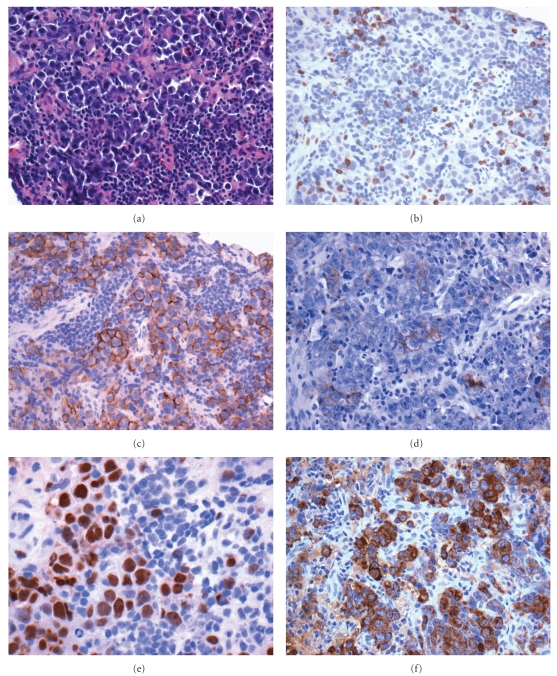
Plasmablastic lymphoma; (a) biopsy of abdominal lesion (hematoxylin and eosin, 200x); (b) LCA stain (200x); (c) CD138 stain (200x); (d) EMA stain (200x); (e) MUM-1 stain (200x); (f) Kappa stain (200x).

HIV-PBL at low power demonstrates diffuse effacement of normal tissue architecture by cells that on higher power are reminiscent of immunoblasts. The cytomorphological features may include eccentric nuclei within eosinophilic to amphophilic granular cytoplasm. The nuclei often demonstrate vesicular chromatin and prominent central nucleoli. Mature B-cell markers (e.g., CD20 or PAX5) are typically negative, with the exception of CD79a and MUM-1 which show variable positivity (see Figure 12 for a selection of stains noted in our unique case of HIV-associated plasmablastic lymphoma). Most importantly, the cells are positive for the plasma cell markers CD38 and CD138 and often show intracytoplasmic immunoglobulin staining. EBV is often positive for EBER by in situ hybridization but, as we have observed in our institution, immunohistochemistry for latent membrane protein may not always stain positive due to its relatively low sensitivity. Of greater importance is the use of HHV-8 staining which is generally negative in plasmablastic lymphoma (and usually positive in primary effusion lymphomas, also known to occur characteristically in the HIV population); this information will be helpful in cases of plasmablastic lymphoma involving body cavities. We recommend including CD56 with CD38 or CD138 stains since, in most cases, positivity for CD56 in this context should raise concerns regarding the possibility of an extramedullary plasmacytoma. It is prudent to also entertain the (often more likely possibility) of a carcinoma with plasmacytoid morphology; melanoma, often called the great imitator, should also be ruled out. As in most cases of suspect lymphoma, flow cytometry is very helpful at demonstrating clonality; though, in most cases of plasmablastic lymphoma, the morphologic features are sufficient to confirm a malignancy.

Outcomes in cases of HIV-PBL appear to have improved since regular treatment with HAART was introduced [9]. Some authors advocate similar chemotherapeutic regimens as those used in cases of HIV-BL [9]. The single case of HIV-PBL encountered at our institution was treated with CHOP, which needed to be rapidly discontinued due to the development of neutropenia; the patient died only days later due to overwhelming sepsis.

## 8. Discussion

HIV/AIDS creates a milieu of combined immunosuppression and antigenic stimulation in lymph nodes. This environment, especially in combination with concurrent infection, creates a permissive environment for the development of a lymphoproliferative disorder. We performed a review of the lymphoproliferative diseases identified in our regional pathology department over the past five years and identified 12 cases demonstrating a lymphoproliferative disorder (amounting to an approximate incidence of 1% of the HIV-positive population). These entities, furthermore, were diverse and spanned much of the spectrum of lymphoproliferative entities known to be associated with HIV.

A number of important themes are appreciated upon review of the literature pertaining to HIV-associated lymphoid lesions. The HIV positive patient population is a unique cohort with an enhanced tendency toward developing lymphoproliferative disorders. HIV-patients are also more prone to developing highly aggressive malignancies than the general population. The use of HAART improves both the risk of and the outcomes in many HIV-associated lymphoproliferative disorders. The inherently immunosuppressed state of HIV makes the choice of chemotherapy difficult and often complicated by infection. The heterogeneity of HIV-associated lymphoproliferative disorders often necessitates extensive ancillary testing, including immunohistochemistry, flow cytometry, and molecular testing as they are often diagnostically challenging. The highly variable prognosis ascribed to the spectrum of HIV-associated lymphoproliferative diseases necessitates diligence and speed in the diagnostic work-up. Finally, clear communication between clinicians and consultant pathologists is paramount at ensuring correct diagnosis and proper patient management.

## Figures and Tables

**Figure 3:**
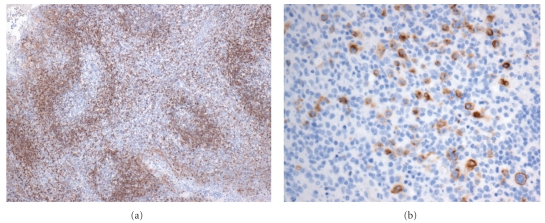
HIV-associated lymphadenopathy. (a) BCL-2 stain demonstrating normal immunophenotype (100x); (b) EBV stain (200x).

**Table 1:** 

Case	Age/sex	Diagnosis/primary site	Followup data
1	38/M	Hodgkin's lymphoma/left axillary lymph node	Lost to followup
2	37/M	Polymorphic lymphoid proliferations resembling posttransplant lymphoproliferative disease/tonsils and lymph node	Lost to followup
3	35/F	Diffuse large B-cell lymphoma/liver	Alive and well
4	49/M	Burkitt's lymphoma/liver	Dead of disease
5	39/M	HIV-associated lymphadenitis/mesenteric lymph node	Alive and well
6	48/M	Burkitt's lymphoma/tonsil	Alive and well
7	47/F	HIV-associated lymphadenitis/axillary lymph node	Alive and well
8	47/M	HIV-associated lymphadenitis/inguinal lymph node	Alive and well
9	58/M	HIV-associated lymphadenitis/inguinal lymph node	Alive and well
10	61/M	Diffuse large B-cell lymphoma/gastric mucosa	Alive and well
11	36/M	Hodgkin's lymphoma/central nervous system	Alive and well
12	42/F	Plasmablastic lymphoma/retroperitoneal mass with concurrent pleural effusion	Dead of disease
